# 4-({[4-Amino-5-(4-chloro­anilinometh­yl)-4*H*-1,2,4-triazol-3-yl]sulfan­yl}acet­yl)-3-(4-meth­oxy­phen­yl)-1,2,3-oxadiazol-3-ium-5-olate

**DOI:** 10.1107/S1600536810046155

**Published:** 2010-11-13

**Authors:** Hoong-Kun Fun, Madhukar Hemamalini, Nithinchandra, Balakrishna Kalluraya

**Affiliations:** a X-ray Crystallography Unit, School of Physics, Universiti Sains Malaysia, 11800 USM, Penang, Malaysia; b Department of Studies in Chemistry, Mangalore University, Mangalagangotri, Mangalore 574 199, India

## Abstract

In the title sydnone compound, C_20_H_18_ClN_7_O_4_S, the oxadiazole, triazole, chloro-substituted and meth­oxy-substituted phenyl rings are essentially planar, with maximum deviations of 0.007 (3), 0.009 (2), 0.017 (2) and 0.002 (3) Å, respectively. The dihedral angles between the chloro-substituted phenyl ring and the triazole ring, the triazole ring and the oxadiazole ring, and the oxadiazole ring and the methoxy-substituted phenyl ring are 80.02 (13), 85.68 (14) and 51.62 (14)°, respectively. In the crystal, mol­ecules are connected *via* inter­molecular N—H⋯N, N—H⋯O and C—H⋯O hydrogen bonds, forming sheets lying parallel to the *ac* plane.

## Related literature

For details and biological applications of sydnones, see: Rai *et al.* (2008 ▶); Jyothi *et al.* (2008 ▶); Kalluraya *et al.* (2002 ▶). For bond-length data, see: Allen *et al.* (1987 ▶). For the stability of the temperature controller used in the data collection, see: Cosier & Glazer (1986 ▶).
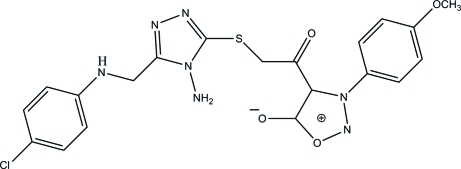


## Experimental

### 

#### Crystal data

C_20_H_18_ClN_7_O_4_S
*M* *_r_* = 487.92
Monoclinic, 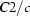

*a* = 20.109 (3) Å
*b* = 5.8952 (8) Å
*c* = 36.369 (5) Å
β = 96.076 (3)°
*V* = 4287.2 (10) Å^3^
*Z* = 8
Mo *K*α radiation
μ = 0.32 mm^−1^
*T* = 100 K
0.40 × 0.13 × 0.04 mm

#### Data collection

Bruker APEXII DUO CCD area-detector diffractometer
Absorption correction: multi-scan (*SADABS*; Bruker, 2009) ▶ *T*_min_ = 0.883, *T*_max_ = 0.989
8905 measured reflections
4429 independent reflections
3255 reflections with *I* > 2σ(*I*)
*R*_int_ = 0.037

#### Refinement

*R*[*F* ^2^ > 2σ(*F* ^2^)] = 0.044
*wR*(*F* ^2^) = 0.132
*S* = 1.08
4429 reflections
311 parameters
H atoms treated by a mixture of independent and constrained refinement
Δρ_max_ = 0.33 e Å^−3^
Δρ_min_ = −0.35 e Å^−3^

### 

Data collection: *APEX2* (Bruker, 2009 ▶); cell refinement: *SAINT* (Bruker, 2009 ▶); data reduction: *SAINT*; program(s) used to solve structure: *SHELXTL* (Sheldrick, 2008 ▶); program(s) used to refine structure: *SHELXTL*; molecular graphics: *SHELXTL*; software used to prepare material for publication: *SHELXTL* and *PLATON* (Spek, 2009 ▶).

## Supplementary Material

Crystal structure: contains datablocks global, I. DOI: 10.1107/S1600536810046155/rz2514sup1.cif

Structure factors: contains datablocks I. DOI: 10.1107/S1600536810046155/rz2514Isup2.hkl

Additional supplementary materials:  crystallographic information; 3D view; checkCIF report

## Figures and Tables

**Table 1:** Hydrogen-bond geometry (Å, °)

*D*—H⋯*A*	*D*—H	H⋯*A*	*D*⋯*A*	*D*—H⋯*A*
N6—H1*N*6⋯N4^i^	0.93 (3)	2.08 (3)	2.947 (3)	155 (2)
N7—H1*N*7⋯O3^ii^	0.86 (3)	2.22 (3)	2.990 (3)	150 (3)
N6—H2*N*6⋯O2^iii^	0.90 (3)	2.15 (3)	2.983 (3)	153 (2)
C4—H4*A*⋯O4^iv^	0.93	2.53	3.337 (3)	145

Symmetry codes: (i) 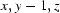; (ii) 
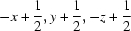
; (iii) 
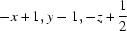
; (iv) 
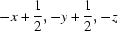
.

**Fig. 1.**
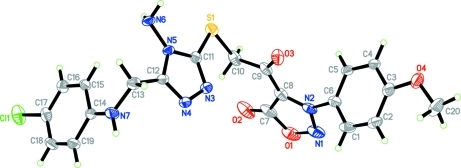
The asymmetric unit of the title compound, showing 50% probability displacement ellipsoids and the atom-numbering scheme.

**Fig. 2.**
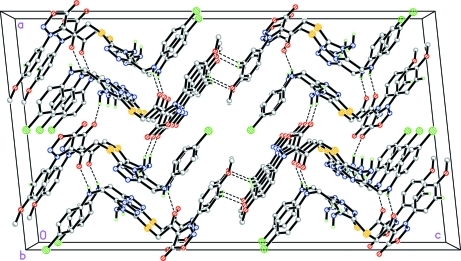
The crystal packing of the title compound, showing hydrogen-bonded (dashed lines) two-dimensional networks parallel to the ac plane.

## supplementary crystallographic information

### Comment

Sydnones are mesoionic heterocyclic aromatic compounds. The study of sydnones still remains a field of interest because of their electronic structures and also because of the varied types of biological activities (Rai *et al.*, 2008). Recently sydnone derivatives were found to exhibit promising antimicrobial properties (Kalluraya *et al.*, 2002). Since their discovery, sydnones have shown diverse biological activities and it is thought that the meso-ionic nature of the sydnone ring promotes significant interactions with biological systems. Because of the wide variety of properties displayed by sydnones, we were prompted to synthesize a new S-substituted triazoles containing a sydnone  ring.  Photochemical bromination of 3-aryl-4-acetylsydnone afforded 3-aryl-4 bromoacetylsydnones.  Condensation of 3-substituted-4-amino-5-mecapto-1,2,4-triazoles with 3-aryl-4-bromoacetylsydnones yielded S-substituted triazoles derivatives (Jyothi *et al.*, 2008).

In the title compound, (Fig. 1), the rings A (C14–C19), B (N3/N4/N5/C11–C12), C (N1/N2/O1/C7–C8) and D (C1–C6) are essentially planar. The dihedral angle between the best planes of the rings are A/B = 80.02 (13)°, A/C = 53.76 (14)°, A/D = 5.24 (12)°, B/C = 85.68 (14)°, B/D = 85.12 (13)° and C/D = 51.62 (14)°.  The bond lengths (Allen *et al.*, 1987) and angles are normal.

In the crystal structure (Fig. 2), the molecules are connected via intermolecular N6—H1N6···N4, N7—H1N7···O3, N6—H2N6···O2 and C4—H4A···O4 hydrogen bonds to form two-dimensional networks parallel to the *ac* plane.

### Experimental

A catalytic amount of anhydrous sodium acetate was added to solution of 4-bromoacetyl-3-(*p*-anisyl)sydnone (0.01 mol) and 4-amino-5-(*p*-chlorophenyl) aminomethyl-4*H*-1,2,4-triazole-3-thiol (0.01 mol) in ethanol. The solution was stirred at room temperature for 2-3 hours. The solid product which separated was filtered and dried. It was then recrystallized from ethanol. Crystals suitable for X-ray analysis were obtained from a mixture of DMF and ethanol (1:2 *v*/*v*) by slow evaporation.

### Refinement

Atoms H1N6 and H2N6 were located in a difference Fourier map and refined freely [N–H = 0.86 (4)–0.92 (3) Å]. The remaining H atoms were positioned geometrically [C–H = 0.93 or 0.96 Å] and were refined using a riding model, with *U*_iso_(H) = 1.2 *U*_eq_(C) or 1.5 *U*_eq_(C) fo methyl H atoms.

### Crystal data

**Table:**

C_20_H_18_ClN_7_O_4_S	*F*(000) = 2016
*M**_r_* = 487.92	*D*_x_ = 1.512 Mg m^−^^3^
Monoclinic, *C*2/*c*	Mo *K*α radiation, λ = 0.71073 Å
Hall symbol: -C 2yc	Cell parameters from 2099 reflections
*a* = 20.109 (3) Å	θ = 3.2–28.1°
*b* = 5.8952 (8) Å	µ = 0.32 mm^−^^1^
*c* = 36.369 (5) Å	*T* = 100 K
β = 96.076 (3)°	Plate, yellow
*V* = 4287.2 (10) Å^3^	0.40 × 0.13 × 0.04 mm
*Z* = 8	

### Data collection

**Table:**

Bruker APEXII DUO CCD area-detector diffractometer	4429 independent reflections
Radiation source: fine-focus sealed tube	3255 reflections with *I* > 2σ(*I*)
graphite	*R*_int_ = 0.037
φ and ω scans	θ_max_ = 26.5°, θ_min_ = 2.0°
Absorption correction: multi-scan (*SADABS*; Bruker, 2009)	*h* = −25→25
*T*_min_ = 0.883, *T*_max_ = 0.989	*k* = −7→7
8905 measured reflections	*l* = −34→45

### Refinement

**Table:**

Refinement on *F*^2^	Primary atom site location: structure-invariant direct methods
Least-squares matrix: full	Secondary atom site location: difference Fourier map
*R*[*F*^2^ > 2σ(*F*^2^)] = 0.044	Hydrogen site location: inferred from neighbouring sites
*wR*(*F*^2^) = 0.132	H atoms treated by a mixture of independent and constrained refinement
*S* = 1.08	*w* = 1/[σ^2^(*F*_o_^2^) + (0.063*P*)^2^ + 1.4574*P*] where *P* = (*F*_o_^2^ + 2*F*_c_^2^)/3
4429 reflections	(Δ/σ)_max_ < 0.001
311 parameters	Δρ_max_ = 0.33 e Å^−^^3^
0 restraints	Δρ_min_ = −0.35 e Å^−^^3^

### Special details

**Table:**

Experimental. The crystal was placed in the cold stream of an Oxford Cryosystems Cobra open-flow nitrogen cryostat (Cosier & Glazer, 1986) operating at 100.0 (1) K.
Geometry. All s.u.'s (except the s.u. in the dihedral angle between two l.s. planes) are estimated using the full covariance matrix. The cell s.u.'s are taken into account individually in the estimation of s.u.'s in distances, angles and torsion angles; correlations between s.u.'s in cell parameters are only used when they are defined by crystal symmetry. An approximate (isotropic) treatment of cell s.u.'s is used for estimating s.u.'s involving l.s. planes.
Refinement. Refinement of F^2^ against ALL reflections. The weighted R-factor wR and goodness of fit S are based on F^2^, conventional R-factors R are based on F, with F set to zero for negative F^2^. The threshold expression of F^2^ > 2σ(F^2^) is used only for calculating R-factors(gt) etc. and is not relevant to the choice of reflections for refinement. R-factors based on F^2^ are statistically about twice as large as those based on F, and R- factors based on ALL data will be even larger.

### Fractional atomic coordinates and isotropic or equivalent isotropic displacement parameters (Å^2^)

**Table:**

	*x*	*y*	*z*	*U*_iso_*/*U*_eq_	
Cl1	0.49815 (4)	0.56466 (15)	0.451234 (19)	0.0463 (2)	
S1	0.41984 (3)	0.08369 (12)	0.219978 (18)	0.02935 (18)	
O1	0.47596 (10)	0.9335 (3)	0.13290 (6)	0.0453 (6)	
O2	0.52595 (10)	0.7437 (4)	0.18278 (6)	0.0484 (6)	
O3	0.35348 (8)	0.3013 (3)	0.15066 (5)	0.0322 (4)	
O4	0.17845 (9)	0.4831 (3)	0.01486 (5)	0.0349 (5)	
N1	0.42244 (13)	0.9057 (4)	0.10652 (7)	0.0404 (6)	
N2	0.39537 (10)	0.7121 (4)	0.11473 (6)	0.0280 (5)	
N3	0.34474 (11)	0.4519 (4)	0.23488 (6)	0.0266 (5)	
N4	0.30066 (10)	0.5097 (4)	0.26070 (5)	0.0251 (5)	
N5	0.33599 (9)	0.1657 (3)	0.27278 (5)	0.0198 (4)	
N6	0.34284 (12)	−0.0438 (4)	0.29125 (6)	0.0254 (5)	
H1N6	0.3216 (13)	−0.156 (5)	0.2766 (7)	0.023 (7)*	
N7	0.26410 (11)	0.5056 (4)	0.33948 (6)	0.0288 (5)	
H1N7	0.2383 (17)	0.621 (6)	0.3370 (9)	0.056 (11)*	
C1	0.28726 (14)	0.8080 (5)	0.08290 (7)	0.0332 (6)	
H1A	0.2903	0.9472	0.0950	0.040*	
C2	0.23260 (13)	0.7609 (5)	0.05768 (7)	0.0299 (6)	
H2A	0.1986	0.8669	0.0526	0.036*	
C3	0.22975 (13)	0.5494 (5)	0.04003 (7)	0.0282 (6)	
C4	0.28062 (13)	0.3919 (4)	0.04767 (6)	0.0260 (6)	
H4A	0.2779	0.2522	0.0357	0.031*	
C5	0.33487 (13)	0.4398 (4)	0.07267 (6)	0.0241 (5)	
H5A	0.3691	0.3347	0.0777	0.029*	
C6	0.33718 (13)	0.6490 (4)	0.09012 (7)	0.0262 (6)	
C7	0.48223 (14)	0.7465 (5)	0.15758 (8)	0.0374 (7)	
C8	0.42757 (12)	0.6031 (5)	0.14469 (7)	0.0290 (6)	
C9	0.40503 (12)	0.3965 (5)	0.16192 (7)	0.0271 (6)	
C10	0.45183 (12)	0.3144 (5)	0.19467 (7)	0.0335 (6)	
H10A	0.4618	0.4406	0.2115	0.040*	
H10B	0.4935	0.2668	0.1859	0.040*	
C11	0.36414 (11)	0.2442 (4)	0.24269 (6)	0.0237 (5)	
C12	0.29609 (11)	0.3372 (4)	0.28250 (6)	0.0206 (5)	
C13	0.25244 (12)	0.3233 (4)	0.31341 (7)	0.0260 (6)	
H13A	0.2059	0.3259	0.3031	0.031*	
H13B	0.2606	0.1801	0.3262	0.031*	
C14	0.32161 (12)	0.5235 (4)	0.36322 (6)	0.0214 (5)	
C15	0.36909 (12)	0.3484 (4)	0.36751 (6)	0.0218 (5)	
H15A	0.3640	0.2210	0.3524	0.026*	
C16	0.42330 (12)	0.3642 (5)	0.39407 (6)	0.0263 (6)	
H16A	0.4544	0.2471	0.3969	0.032*	
C17	0.43145 (13)	0.5526 (5)	0.41640 (7)	0.0285 (6)	
C18	0.38641 (14)	0.7303 (5)	0.41185 (7)	0.0315 (6)	
H18A	0.3927	0.8589	0.4266	0.038*	
C19	0.33229 (13)	0.7165 (4)	0.38544 (7)	0.0300 (6)	
H19A	0.3024	0.8370	0.3823	0.036*	
C20	0.12571 (15)	0.6392 (6)	0.00442 (8)	0.0426 (8)	
H20A	0.0941	0.5708	−0.0139	0.064*	
H20B	0.1438	0.7737	−0.0056	0.064*	
H20C	0.1037	0.6783	0.0257	0.064*	
H2N6	0.3874 (13)	−0.066 (4)	0.2955 (7)	0.018 (6)*	

### Atomic displacement parameters (Å^2^)

**Table:**

	*U*^11^	*U*^22^	*U*^33^	*U*^12^	*U*^13^	*U*^23^
Cl1	0.0354 (4)	0.0690 (6)	0.0335 (4)	−0.0181 (4)	−0.0004 (3)	−0.0100 (4)
S1	0.0262 (3)	0.0321 (4)	0.0290 (3)	0.0061 (3)	−0.0005 (3)	−0.0056 (3)
O1	0.0459 (12)	0.0328 (12)	0.0607 (14)	−0.0217 (10)	0.0229 (11)	−0.0162 (11)
O2	0.0299 (11)	0.0598 (15)	0.0570 (13)	−0.0188 (11)	0.0115 (10)	−0.0279 (12)
O3	0.0285 (10)	0.0403 (11)	0.0269 (9)	−0.0176 (9)	−0.0010 (7)	0.0009 (8)
O4	0.0395 (11)	0.0384 (11)	0.0266 (9)	0.0133 (9)	0.0019 (8)	0.0056 (9)
N1	0.0466 (15)	0.0270 (13)	0.0518 (15)	−0.0160 (12)	0.0246 (12)	−0.0100 (12)
N2	0.0323 (12)	0.0208 (11)	0.0340 (11)	−0.0107 (10)	0.0181 (9)	−0.0083 (10)
N3	0.0317 (12)	0.0215 (11)	0.0256 (11)	0.0039 (10)	−0.0010 (9)	0.0010 (9)
N4	0.0309 (11)	0.0180 (10)	0.0252 (10)	0.0023 (9)	−0.0022 (9)	−0.0002 (9)
N5	0.0198 (10)	0.0152 (10)	0.0228 (9)	0.0014 (8)	−0.0047 (8)	0.0006 (8)
N6	0.0299 (12)	0.0157 (11)	0.0287 (11)	0.0060 (10)	−0.0061 (9)	0.0027 (9)
N7	0.0310 (12)	0.0256 (12)	0.0297 (12)	0.0120 (11)	0.0030 (9)	−0.0034 (10)
C1	0.0452 (16)	0.0207 (13)	0.0380 (14)	0.0020 (13)	0.0245 (13)	−0.0018 (12)
C2	0.0354 (14)	0.0233 (13)	0.0335 (13)	0.0080 (12)	0.0155 (12)	0.0069 (12)
C3	0.0364 (14)	0.0297 (14)	0.0203 (12)	0.0029 (12)	0.0119 (11)	0.0058 (11)
C4	0.0393 (15)	0.0182 (12)	0.0217 (12)	0.0041 (11)	0.0083 (10)	0.0005 (10)
C5	0.0325 (13)	0.0168 (12)	0.0244 (12)	0.0007 (11)	0.0089 (10)	0.0021 (10)
C6	0.0316 (14)	0.0214 (13)	0.0275 (12)	−0.0037 (11)	0.0124 (10)	−0.0015 (11)
C7	0.0308 (14)	0.0361 (16)	0.0482 (16)	−0.0155 (13)	0.0181 (13)	−0.0211 (14)
C8	0.0248 (13)	0.0319 (15)	0.0318 (13)	−0.0120 (12)	0.0105 (11)	−0.0107 (12)
C9	0.0238 (13)	0.0324 (15)	0.0261 (12)	−0.0059 (12)	0.0077 (10)	−0.0105 (11)
C10	0.0226 (13)	0.0460 (17)	0.0317 (13)	−0.0079 (13)	0.0017 (11)	−0.0106 (13)
C11	0.0225 (12)	0.0251 (13)	0.0218 (12)	0.0035 (11)	−0.0055 (10)	−0.0030 (10)
C12	0.0199 (12)	0.0161 (12)	0.0240 (12)	0.0011 (10)	−0.0061 (9)	−0.0018 (10)
C13	0.0232 (12)	0.0245 (13)	0.0294 (13)	0.0033 (11)	−0.0020 (10)	0.0007 (11)
C14	0.0259 (12)	0.0189 (12)	0.0206 (11)	−0.0010 (10)	0.0080 (9)	0.0006 (10)
C15	0.0250 (12)	0.0194 (12)	0.0217 (11)	−0.0010 (10)	0.0055 (9)	−0.0023 (10)
C16	0.0231 (12)	0.0306 (14)	0.0261 (12)	0.0013 (11)	0.0074 (10)	−0.0004 (11)
C17	0.0272 (13)	0.0364 (16)	0.0226 (12)	−0.0116 (12)	0.0060 (10)	−0.0025 (11)
C18	0.0445 (16)	0.0230 (14)	0.0290 (13)	−0.0083 (13)	0.0139 (12)	−0.0085 (11)
C19	0.0414 (15)	0.0210 (13)	0.0299 (13)	0.0047 (12)	0.0141 (12)	−0.0015 (11)
C20	0.0397 (16)	0.0508 (19)	0.0381 (16)	0.0198 (15)	0.0080 (13)	0.0162 (15)

### Geometric parameters (Å, °)

**Table:**

Cl1—C17	1.746 (3)	C2—H2A	0.9300
S1—C11	1.740 (2)	C3—C4	1.388 (4)
S1—C10	1.799 (3)	C4—C5	1.374 (4)
O1—N1	1.373 (3)	C4—H4A	0.9300
O1—C7	1.418 (4)	C5—C6	1.385 (3)
O2—C7	1.201 (3)	C5—H5A	0.9300
O3—C9	1.211 (3)	C7—C8	1.426 (4)
O4—C3	1.362 (3)	C8—C9	1.463 (4)
O4—C20	1.425 (3)	C9—C10	1.517 (4)
N1—N2	1.312 (3)	C10—H10A	0.9700
N2—C8	1.368 (3)	C10—H10B	0.9700
N2—C6	1.445 (3)	C12—C13	1.500 (3)
N3—C11	1.307 (3)	C13—H13A	0.9700
N3—N4	1.400 (3)	C13—H13B	0.9700
N4—C12	1.298 (3)	C14—C19	1.398 (3)
N5—C12	1.361 (3)	C14—C15	1.404 (3)
N5—C11	1.365 (3)	C15—C16	1.381 (3)
N5—N6	1.406 (3)	C15—H15A	0.9300
N6—H1N6	0.92 (3)	C16—C17	1.375 (4)
N6—H2N6	0.90 (3)	C16—H16A	0.9300
N7—C14	1.372 (3)	C17—C18	1.383 (4)
N7—C13	1.436 (3)	C18—C19	1.376 (4)
N7—H1N7	0.86 (4)	C18—H18A	0.9300
C1—C6	1.378 (4)	C19—H19A	0.9300
C1—C2	1.383 (4)	C20—H20A	0.9600
C1—H1A	0.9300	C20—H20B	0.9600
C2—C3	1.401 (4)	C20—H20C	0.9600
			
C11—S1—C10	96.59 (13)	C8—C9—C10	114.0 (2)
N1—O1—C7	111.1 (2)	C9—C10—S1	114.66 (18)
C3—O4—C20	118.9 (2)	C9—C10—H10A	108.6
N2—N1—O1	105.0 (2)	S1—C10—H10A	108.6
N1—N2—C8	114.6 (2)	C9—C10—H10B	108.6
N1—N2—C6	114.3 (2)	S1—C10—H10B	108.6
C8—N2—C6	131.0 (2)	H10A—C10—H10B	107.6
C11—N3—N4	106.1 (2)	N3—C11—N5	110.6 (2)
C12—N4—N3	108.1 (2)	N3—C11—S1	126.8 (2)
C12—N5—C11	105.2 (2)	N5—C11—S1	122.58 (19)
C12—N5—N6	124.0 (2)	N4—C12—N5	110.0 (2)
C11—N5—N6	130.8 (2)	N4—C12—C13	125.8 (2)
N5—N6—H1N6	109.7 (16)	N5—C12—C13	124.2 (2)
N5—N6—H2N6	104.7 (16)	N7—C13—C12	112.7 (2)
H1N6—N6—H2N6	113 (2)	N7—C13—H13A	109.0
C14—N7—C13	122.7 (2)	C12—C13—H13A	109.0
C14—N7—H1N7	118 (2)	N7—C13—H13B	109.0
C13—N7—H1N7	118 (2)	C12—C13—H13B	109.0
C6—C1—C2	120.1 (2)	H13A—C13—H13B	107.8
C6—C1—H1A	120.0	N7—C14—C19	119.6 (2)
C2—C1—H1A	120.0	N7—C14—C15	122.1 (2)
C1—C2—C3	118.3 (2)	C19—C14—C15	118.2 (2)
C1—C2—H2A	120.9	C16—C15—C14	120.3 (2)
C3—C2—H2A	120.9	C16—C15—H15A	119.8
O4—C3—C4	115.6 (2)	C14—C15—H15A	119.8
O4—C3—C2	123.7 (2)	C17—C16—C15	120.2 (2)
C4—C3—C2	120.7 (2)	C17—C16—H16A	119.9
C5—C4—C3	120.8 (2)	C15—C16—H16A	119.9
C5—C4—H4A	119.6	C16—C17—C18	120.4 (2)
C3—C4—H4A	119.6	C16—C17—Cl1	119.7 (2)
C4—C5—C6	118.2 (2)	C18—C17—Cl1	119.9 (2)
C4—C5—H5A	120.9	C19—C18—C17	119.9 (2)
C6—C5—H5A	120.9	C19—C18—H18A	120.1
C1—C6—C5	122.0 (2)	C17—C18—H18A	120.1
C1—C6—N2	117.9 (2)	C18—C19—C14	120.9 (2)
C5—C6—N2	119.9 (2)	C18—C19—H19A	119.6
O2—C7—O1	120.2 (3)	C14—C19—H19A	119.6
O2—C7—C8	136.0 (3)	O4—C20—H20A	109.5
O1—C7—C8	103.8 (2)	O4—C20—H20B	109.5
N2—C8—C7	105.5 (2)	H20A—C20—H20B	109.5
N2—C8—C9	126.2 (2)	O4—C20—H20C	109.5
C7—C8—C9	128.0 (3)	H20A—C20—H20C	109.5
O3—C9—C8	122.3 (2)	H20B—C20—H20C	109.5
O3—C9—C10	123.7 (2)		
			
C7—O1—N1—N2	1.3 (3)	C7—C8—C9—C10	−8.4 (4)
O1—N1—N2—C8	−1.1 (3)	O3—C9—C10—S1	−8.0 (3)
O1—N1—N2—C6	179.88 (19)	C8—C9—C10—S1	172.08 (18)
C11—N3—N4—C12	0.3 (3)	C11—S1—C10—C9	−75.5 (2)
C6—C1—C2—C3	0.0 (4)	N4—N3—C11—N5	−1.3 (3)
C20—O4—C3—C4	−177.4 (2)	N4—N3—C11—S1	−179.44 (17)
C20—O4—C3—C2	2.8 (3)	C12—N5—C11—N3	1.7 (3)
C1—C2—C3—O4	179.7 (2)	N6—N5—C11—N3	−179.1 (2)
C1—C2—C3—C4	−0.1 (4)	C12—N5—C11—S1	179.98 (16)
O4—C3—C4—C5	−179.9 (2)	N6—N5—C11—S1	−0.9 (3)
C2—C3—C4—C5	0.0 (4)	C10—S1—C11—N3	13.6 (2)
C3—C4—C5—C6	0.3 (4)	C10—S1—C11—N5	−164.31 (19)
C2—C1—C6—C5	0.2 (4)	N3—N4—C12—N5	0.8 (3)
C2—C1—C6—N2	175.8 (2)	N3—N4—C12—C13	−177.9 (2)
C4—C5—C6—C1	−0.4 (4)	C11—N5—C12—N4	−1.5 (3)
C4—C5—C6—N2	−175.9 (2)	N6—N5—C12—N4	179.3 (2)
N1—N2—C6—C1	−50.1 (3)	C11—N5—C12—C13	177.2 (2)
C8—N2—C6—C1	131.1 (3)	N6—N5—C12—C13	−2.0 (3)
N1—N2—C6—C5	125.5 (2)	C14—N7—C13—C12	−70.2 (3)
C8—N2—C6—C5	−53.3 (3)	N4—C12—C13—N7	−55.2 (3)
N1—O1—C7—O2	−179.0 (2)	N5—C12—C13—N7	126.3 (2)
N1—O1—C7—C8	−1.0 (3)	C13—N7—C14—C19	174.1 (2)
N1—N2—C8—C7	0.5 (3)	C13—N7—C14—C15	−9.4 (4)
C6—N2—C8—C7	179.3 (2)	N7—C14—C15—C16	−174.1 (2)
N1—N2—C8—C9	174.0 (2)	C19—C14—C15—C16	2.5 (3)
C6—N2—C8—C9	−7.1 (4)	C14—C15—C16—C17	−0.4 (4)
O2—C7—C8—N2	177.8 (3)	C15—C16—C17—C18	−1.6 (4)
O1—C7—C8—N2	0.3 (3)	C15—C16—C17—Cl1	177.12 (18)
O2—C7—C8—C9	4.4 (5)	C16—C17—C18—C19	1.5 (4)
O1—C7—C8—C9	−173.1 (2)	Cl1—C17—C18—C19	−177.23 (19)
N2—C8—C9—O3	−0.4 (4)	C17—C18—C19—C14	0.6 (4)
C7—C8—C9—O3	171.7 (3)	N7—C14—C19—C18	174.1 (2)
N2—C8—C9—C10	179.5 (2)	C15—C14—C19—C18	−2.6 (3)

### Hydrogen-bond geometry (Å, °)

**Table:**

*D*—H···*A*	*D*—H	H···*A*	*D*···*A*	*D*—H···*A*
N6—H1N6···N4^i^	0.93 (3)	2.08 (3)	2.947 (3)	155 (2)
N7—H1N7···O3^ii^	0.86 (3)	2.22 (3)	2.990 (3)	150 (3)
N6—H2N6···O2^iii^	0.90 (3)	2.15 (3)	2.983 (3)	153 (2)
C4—H4A···O4^iv^	0.93	2.53	3.337 (3)	145

Symmetry codes: (i) *x*, *y*−1, *z*; (ii) −*x*+1/2, *y*+1/2, −*z*+1/2; (iii) −*x*+1, *y*−1, −*z*+1/2; (iv) −*x*+1/2, −*y*+1/2, −*z*.
